# High definition bronchoscopy: a randomized exploratory study of diagnostic value compared to standard white light bronchoscopy and autofluorescence bronchoscopy

**DOI:** 10.1186/s12931-015-0193-7

**Published:** 2015-03-07

**Authors:** Erik HFM van der Heijden, Wouter Hoefsloot, Hieronymus WH van Hees, Olga CJ Schuurbiers

**Affiliations:** Department of Pulmonary Diseases, Radboud University Medical Center, Nijmegen, The Netherlands

## Abstract

**Background:**

Videobronchoscopy is an essential diagnostic procedure for evaluation of the central airways and pivotal for the diagnosis and staging of lung cancer. Technological improvements have resulted in high definition (HD) images with advanced real time image enhancement techniques (i-scan).

**Objectives:**

In this study we aimed to explore the sensitivity of HD+ i-scan bronchoscopy for detection of epithelial changes like vascular abnormalities and suspicious preinvasive lesions, and tumors.

**Methods:**

In patients scheduled for a therapeutic or diagnostic procedure under general anesthesia videos of the bronchial tree were made using 5 videobronchoscopy modes in random order: normal white light videobronchoscopy (WLB), HD-bronchoscopy (HD), HD bronchoscopy with surface enhancement technique (i-scan1), HD with surface- and tone enhancement technique (i-scan2) and dual mode autofluorescence videobronchoscopy (AFB). The videos were scored in random order by two independent and blinded expert bronchoscopists.

**Results:**

In 29 patients all videos were available for analysis. Vascular abnormalities were scored most frequently in HD + i-scan2 bronchoscopy (1.33 ± 0.29 abnormal or suspicious sites per patient) as compared to 0.12 ± 0.05 site for AFB (P = 0.003). Sites suspicious for preinvasive lesions were most frequently reported using AFB (0.74 ± 0.12 sites per patient) as compared to 0.17 ± 0.06 for both WLB and HD bronchoscopy (P = 0.003). Tumors were detected equally by all modalities. The preferred modality was HD bronchoscopy with i-scan (tone- plus surface and surface enhancement in respectively 38% and 35% of cases P = 0.006).

**Conclusions:**

This study shows that high definition bronchoscopy with image enhancement technique may result in better detection of subtle vascular abnormalities in the airways. Since these abnormalities may be related to preneoplastic lesions and tumors this is of clinical relevance. Further investigations using this technique relating imaging to histology are warranted.

## Background

Bronchoscopy is one of the most important procedures in diagnosis of lung cancer and other pulmonary diseases. This procedure not only aims to obtain a diagnosis but also renders important anatomical information, subtle changes in the airway epithelium and vascular patterns of the bronchial tree are clues to guide the endoscopist. These subtle changes may influence the choice of treatment, site of biopsy and resectability of cancers when determining resection margins especially in case of centrally located lung cancer but also in case of multifocal premalignant disease. A meta-analysis showed diagnostic superiority of autofluorescence bronchoscopy (AFB) over routine white light bronchoscopy (WLB) in detecting premalignant lesions [1,2]. The superiority of AFB over WLB is especially significant in older studies using fiber optic endoscopes, and the use of video endoscopes further improved detection [3-5]. Yet AFB is not widely used and available in specialized centers only. Furthermore, the latest version of American College of Chest Physicians (ACCP) guideline does not strongly advocate its use but recommends using AF ‘when available’ [6]. This weak advice is mainly due to uncertainty on the natural history and the risk of progression from premalignant lesions to invasive carcinoma and the high rate of false positive findings that need biopsy [6]. Currently, normal video white light bronchoscopy is the standard in most countries and video-autofluorescence bronchoscopy (AFB) is offered by specialized centers only.

Alternatively, the use of filters transmitting only part of the reflected white light (narrow band imaging, NBI) has been shown to be of additional value to detect angiogenic squamous dysplasia (as a precursor for invasive tumors) [7-9] and was reported to be approximately equal to (video-)AFB in terms of sensitivity and slightly better specificity [10,11]. The type of vascular pattern was also related to histological subtype (dotted vessels related to adenocarcinoma and abrupt vessel endings to squamous carcinoma) [12].

Through technological improvement new techniques have become available in the form of high-definition (HD-) bronchoscopy. Furthermore, in combination with improved video processor unit this HD bronchoscope offers real time image enhancement (i-scan Technology) [13]. The impact of this development with high-definition videobronchoscopy using a 1.1 megapixel chip on the diagnostic performance of bronchoscopy is however unknown.

In this exploratory study we therefore aim to compare the sensitivity of HD bronchoscopy, with or without surface enhancement or tone enhancement to video-AFB (the ‘gold standard’ for preinvasive lesions) and standard WLB for detecting abnormalities of the tracheobronchial tree in patients with known or suspected lung cancer, centrally located pulmonary metastatic disease or head and neck cancer.

## Methods

This study is a randomized observational exploratory study with a blinded post procedure analysis of the diagnostic performance of HD bronchoscopy with or without i-scan image enhancement technology in comparison to standard WLB and video AFB.

### Study population

Patients scheduled for diagnostic or therapeutic procedure under general anesthesia were eligible for this exploratory study. Ineligibility criteria are known contraindications for diagnostic bronchoscopy (bleeding disorders, indication for use of anticoagulant therapy (acenocoumarol, warfarine, therapeutic dose of low molecular weight heparine or clopidrogel); known allergy for lidocaine; known pulmonary hypertension; recent and/or uncontrolled cardiac disease; the presence of contraindications for the use of laryngeal mask (anatomical abnormalities, increased risk for intubation (malampatti score 4)); ASA classification greater than or equal to 4; age under 18 years or pregnancy.

### Study procedure

Prior to the scheduled procedure the bronchoscopy studies were performed by an experienced chest physician through a laryngeal mask under general anaesthesia. For bronchoscopic evaluation five different imaging modes were used for evaluation of the central airways in a standardized order. After each evaluation the tip of the bronchoscope was retracted above the vocal cords. The order of the different modes was randomized to prevent bias due to scope or coughing induced epithelial lesions and to prevent operator bias. The five imaging modes used in this study are: Standard white light videobronchoscopy (WLB, using Pentax EB1570 in combination with Pentax EPKi7000 videoprocessor in basic setting); High Definition (HD-) bronchoscopy (HD, using Pentax EB1990i with EPKi-7000 videoprocessor in basic setting); HD-bronchoscopy + surface enhancement (i-scan 1: using Pentax EB1990i and EPKi-7000 with settings brightness +0 average, redness 0, enhancement level +4 surface enhancement +4, tone enhancement off, color enhancement off, noise reduction low); HD-bronchoscopy + tone enhancement (i-scan2: using Pentax EB1990i and EPKi-7000 with settings brightness +1 average, redness 0, enhancement level +4 surface enhancement +4, tone enhancement colon, color enhancement off, noise reduction low) and Auto Fluorescence Bronchoscopy (AFB – using Pentax EB1970A) in dual video mode (simultaneous image of xenon white light and autofluorescence light source with a laser at wavelength 408 nm and laser power 20 to 40 mW in a Pentax SAFE-3000 videoprocessor). Depending on the randomization order, changing of the bronchoscopes and videoprocessors was needed.

High-definition digital videos were made from all procedures without in screen indications of date, time or reference to patient identification. Clinically relevant newly discovered abnormalities were biopsied after completion of the study protocol upon decision of the operator.

The HD-digital video’s were reviewed separately by two experienced bronchoscopists (WH and OS) in random order (mixing all possible modalities and patients) and blinded for patient information and outcome and study date and scored using a predefined scoring system to count vascular abnormal and sites suspicious preinvasive lesions and tumors sites using the scoring system described in earlier studies with AFB and vascular patterns [3,4,9,10,14]. Sites characterized by subtle vascular changes were classified as suspicious following criteria by Herth and Shibuya et al. [9,10] whereas sites suspected for preinvasive lesions were classified based on mucosal irregularities and in case of AFB loss of autofluorescence at the AF image with or without apparent abnormalities on the twin mode WLB of the SAFE300 system as described by Divisi, Haussinger and Ikeda et al. [3,4,14]. Average scores of the total number of abnormal and suspicious sites per patient were used for further analysis. A joined reading was performed with disclosure of the diagnosis in order to determine the subjectively preferred imaging modality.

This study was performed at Radboud University Medical Center, a tertiary care university referral center between December 2012 and October 2013. This study was approved by the RadboudUMC Medical Ethical committee under number 2011/554 and registered on clinicaltrials.gov with identifier: NCT01676012.

### i-scan image enhancement technology

In this study i-scan surface enhancement and tone enhancement imaging were used with the above mentioned settings and decribed by Kodashima and Fujishiro [13]. Surface enhancement is obtained real life by enhancing the luminance intensity of pixels to surrounding pixels with an adapted noise erasure algorithm. This results in an enhancement of edge accentuating of minute structural differences. Tone enhancement is obtained by disintegrating the RGB components of the endoscope image into separate components and reconstruct the image with converted tone curves [13].

### Statistics

Repeated measurements analysis was used to compare the mean number of registered sites for each modality with LSD posthoc testing and Chi-Square for the preferred modality. A P value of < 0.05 is considered statistically significant. Statistical analysis was performed with SPSS 20.0 software (IBM SPSS, Chicago, Il).

## Results

**Figure 1:**
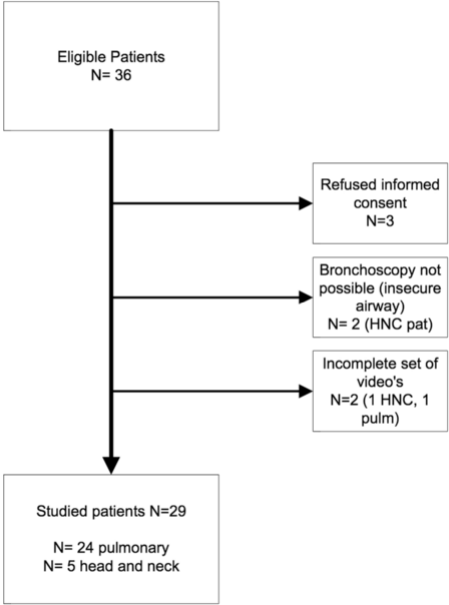
**Consort flow chart of patients included the study.** HNC: head and neck cancer referral patients.

**Figure 2:**
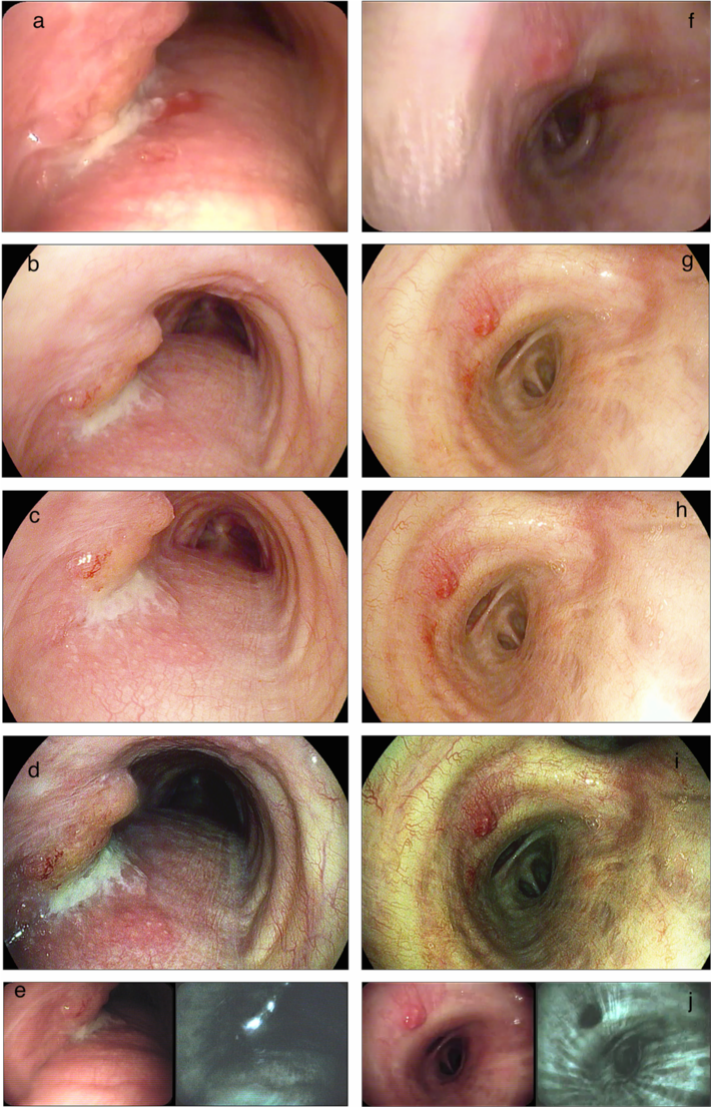
**Representative bronchoscopy images from two patients using (from top to bottom) normal white light videobronchoscopy (WLB, panels a and f); HD-bronchoscopy (panels b and g); HD+ i-scan 1: HD bronchoscopy with surface enhancement (panels c and h); HD+ i-scan2: HD bronchoscopy with surface- and tone enhancement (panels d and i) and autofluorescence bronchoscopy (AFB) in twin mode with WLB image on the left and AFB on the right (panels e and j).** The bronchoscopic images on the left are from a patient with a (recurrent) squamous cell non small cell lung carcinoma in the mid trachea on the left lateral wall. The images on the on the right show a abnormal vascular pattern. Pathology from this site showed squamous metaplasia, some fibrosis and signs of active inflammation.

Of the 36 patients eligible for this study, bronchoscopy was performed in 31 patients and a complete set of videos was available for analysis in 29 patients (Figure 1). The mean age of the study population was 63 years (range 40 – 93 yr), 39% were females. The majority of the investigated patients were referred for an interventional pulmonary endoscopic treatment or diagnostic procedure (n = 24), all others were referred for a surgical procedure by the head and neck surgeon. Of the analyzed patients non-small cell lung cancer (NSCLC) was the most frequent condition (n = 16; 52%); five were referred for a head and neck diagnostic procedure (16%) and one for a suspected combined NSCLC and laryngeal cancer. The remaining patients were scheduled for a interventional pulmonary procedure for metastatic colorectal cancer (n = 2), metastatic renal cell cancer (n = 2), melanoma (n = 1), lymphoma (n = 1) benign tracheal stenosis (n = 2) and sarcoidosis (n = 1). In one of the patients with a head and neck cancer a clinically relevant new lesion was detected that needed biopsy which showed NSCLC. Representative bronchoscopy images indicating the differences between the used bronchoscopy modalities is presented in Figure 2.

### Number of vascular abnormalities detected

**Figure 3:**
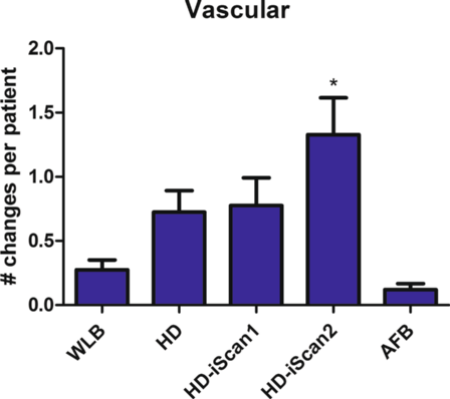
**Number of sites with abnormal and suspicious vascular changes.** WLB: standard white light bronchoscopy; HD: High Definition bronchoscopy; i-scan1: HD bronchoscopy with surface enhancement; i-scan2: HD bronchoscopy with tone enhancement; AFB: autofluorescence video bronchoscopy twin mode (dual image SAFE3000).

Using the different modalities for bronchoscopy the number of vascular abnormal and suspicious lesions detected varied from 0.12 ± 0.05 for AFB to 1.33 ± 0.29 for i-scan2 (P = 0.003, Table 1, Figure 3). Using normal WLB 0.28 ± 0.08 vascular abnormal sites were detected and HD and i-scan1 detected 0.72 ± 0.17 and 0.78 ± 0.22 lesions respectively.

**Table 1:** **Number of sites detected (average per patient)**

	**Vascular**	**Suspicious preinvasive**	**Tumor**
**Modality**			
**WLB**	0,28±0,08	0,17±0,06	1,22±0,28
**HD**	0,72±0,17	0,17±0,06	1,05±0,21
**HD-i-scan1**	0,78±0,22	0,24±0,07	1,07±0,24
**HD-i-scan2**	1,33±0,29*	0,21±0,06	1,03±0,22
**AFB**	0,12±0,05	0,74±0,12*	1,00±0,23
P	*0.003	*0.001	0.488

*significant P value.

### Number of (suspected) preinvasive lesions detected

**Figure 4:**
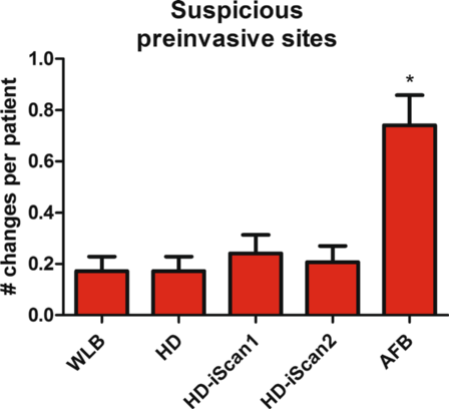
**Number of sites with suspicious preinvasive lesions.** WLB: standard white light bronchoscopy; HD: High Definition bronchoscopy; i-scan1: HD bronchoscopy with surface enhancement; i-scan2: HD bronchoscopy with tone enhancement; AFB: autofluorescence video bronchoscopy dual image mode (SAFE3000).

The number of sites abnormal and suspected sites for (possible) preinvasive lesions using the different modalities for bronchoscopy ranged from 0.17 ± 0.06 for both WLB and HD bronchoscopy to 0.74 ± 0.12 for AFB (P = 0.001, Table 1, Figure 4). Using normal i-scan1 and i-scan 2 0.24 ± 0.07 and 0.21 ± 0.06 possible preinvasive lesions were detected respectively.

### Number of tumor sites detected

The average number of tumor sites detected per patient did not significantly differ between bronchoscopy modes and ranged from 1.0 ± 0.22 in AFB to 1.22 ± 0.28 for WLB. Using the other modalities also approximately 1 tumor site in each patient was discovered (P = 0.488, Table 1).

### Preferred modality

**Figure 5:**
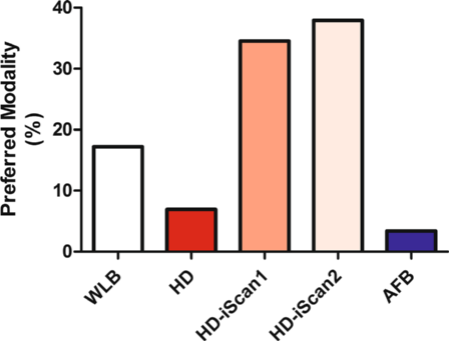
**Preferred modality determined in joined reading expressed as percentage of total.** WLB: standard white light bronchoscopy; HD: High Definition bronchoscopy; i-scan1: HD bronchoscopy with surface enhancement; i-scan2: HD bronchoscopy with tone enhancement; AFB: autofluorescence video bronchoscopy dual image mode (SAFE3000).

In a joint reading with disclosure of the medical outcome the preferred modality was HD bronchoscopy with i-scan image enhancement (P = 0.006). HD + i-scan 2 was preferred in 38% of the cases and HD + i-scan 1 was the preferred modality in 35% of the cases (Figure 5).

## Discussion

This randomized and blinded prospective exploratory study indicates that the superior image quality of HD bronchoscopy, especially in combination with i-scan image enhancement may improve the detection of vascular abnormalities. For possible preinvasive lesions AFB seems to be preferred over normal WLB and HD-bronchoscopy but in this exploratory study no routine biopsies were obtained for histological confirmation. For the detection of endobronchial tumor sites no differences were observed. These results warrant further investigation with histological confirmation of lesions with abnormal vascular patterns.

This study shows that the use of i-scan image enhancement technology in combination with HD bronchoscopy significantls improves detection of vascular changes especially with i-scan2 offering the combination of surface enhancement and tone enhancement rendering a sharp and naturally colored image of the bronchial tree. Vascular changes have been shown to be correlated to angiogenic squamous metaplasia and lung adenocarcinoma or squamous carcinoma, as was reported earlier in studies using the narrow band filtering image technique (NBI) [7-12]. Therefore facilitation of detection of these subtle changes is of clinical importance. It is likely that improvement of the image quality and additional image enhancement technology may improve the overall diagnostic yield of bronchoscopy and improve staging depending on the specificity and percentage of false positive findings in future studies. Furthermore better imaging may reduce procedure time when these techniques become widely available in bronchoscopes designed for everyday use with adequate working channel diameters.

Additionally, it may be possible that these improved imaging techniques may further reduce the need for AFB. This study indicates that AFB may still be the most sensitive modality for detecting sites suspicious for preinvasive lesions by the lack of autofluorescence in combination with normal WLB indicating thickening of the epithelial layer, but since there was no histological confirmation, this result must be interpreted with great caution. In this study AFB was used in the dual video (“twin-mode”) setting offering direct comparison between WLB and AFB. The readers are experienced and trained to detect lesions that lack autofluorescence without gross changes on the WLB image whereas in the other modalities only irregularity of the bronchial mucosa could be classified as suspicious preinvasive lesions. For daily clinical use AFB is only limited available and has shown to have a low specificity with false positive imaging up to one third of the detected lesions [2]. For AFB a dedicated (laser) light source is needed emitting a light at a specific wave length, usually at the 390–440 nm range. This additional technology requirement in combination with decreasing prevalence of centrally located tumors has probably played a role in its low availability in clinical practice. Therefore the use of a single light source (emitting full spectrum white light) in combination with filtering techniques like NBI or post processing real time image enhancement techniques like i-scan will be much easier to implement into daily clinical practice. Furthermore recent evidence comparing AFB to NBI has shown equal sensitivity and slightly better specificity favoring NBI over AFB [10,11]. This is supported by our finding that the preferred image modality in this study was the HD-bronchoscopy with either i-scan settings based on joined reading. Apparently a more natural colored image is preferred over the blue images from AFB even by experienced bronchoscopist.

## Conclusion

This exploratory study shows that high definition bronchoscopy with image enhancement technique may result in better detection of subtle vascular abnormalities in the airways. Since these abnormalities may be related to preneoplastic lesions and tumors this is of clinical relevance. Further investigations using this technique relating imaging to histology are warranted.

## Abbreviations

ACCP: American College of chest physicians
AFB: Autofluorescence bronchoscopy
ASA: American society of anesthesiologist
HD: High definition
HNC: Head and neck cancer
NBI: Narrow band imaging
NSCLC: Non small cell lung cancer
RGB: Red, green, blue
WLB: Normal white light video bronchoscopy
